# Association between periodontitis and vascular endothelial function using noninvasive medical device—A pilot study

**DOI:** 10.1002/cre2.312

**Published:** 2020-07-31

**Authors:** Takahito Fujitani, Norio Aoyama, Fumihiko Hirata, Masato Minabe

**Affiliations:** ^1^ Division of Periodontology, Department of Oral Interdisciplinary Medicine, Graduate School of Dentistry Kanagawa Dental University Yokosuka Japan; ^2^ Yokosuka Tower Clinic Yokosuka Japan

**Keywords:** endothelial dysfunction, noncommunicable disease, periodontal disease

## Abstract

The present study aimed to assess the relationship between periodontal condition and endothelial function using a noninvasive device. Many recent studies have reported associations between periodontitis and cardiovascular diseases. Endothelial dysfunction is the first step of atherosclerosis, but information on the association between periodontal disease and endothelial dysfunction remains limited. Thirty‐three subjects were recruited from among patients at a private medical clinic. We examined vascular endothelial function using a noninvasive medical device and periodontal measurements including probing pocket depth, attachment level, tooth mobility, and oral cleaning condition. Subjects were divided into two groups according to endothelial function score. Tooth mobility and number of lost teeth were increased in the group with endothelial dysfunction. A greater frequency of elderly subjects and altered hemoglobin A1c levels were seen in the endothelial dysfunction group. On multiple logistic regression analysis, increased tooth mobility was independently associated with endothelial dysfunction. Increased tooth mobility, a major periodontal parameter, appears related to endothelial dysfunction.

## 1 INTRODUCTION

In recent years, a relationship between periodontitis and atherosclerosis has been identified. Inflammation plays a fundamental role in the pathogenesis of vascular diseases, and low‐grade chronic inflammation has been shown to be associated with adverse cardiovascular outcomes (Libby, Ridker, & Maseri, 2002). Periodontitis, a common chronic infection of the tissues surrounding teeth, is associated with elevated levels of C‐reactive protein and inflammatory biomarkers (Loos, Craandijk, Hoek, Wertheim‐van Dillen, & van der Velden, 2000; Noack et al., 2001; Slade et al., 2003; Slade, Offenbacher, Beck, Heiss, & Pankow, 2000). Periodontitis involves bacterial infection with Gram‐negative bacteria that invade the superficial and deeper gingival tissues depending on severity (Pihlstrom, Michalowicz, & Johnson, 2005). Previous studies have shown that periodontitis is linked to endothelial dysfunction (Amar et al., 2003), atherosclerosis (Beck et al., 2001), and an increased risk of myocardial infarction (Scannapieco, Bush, & Paju, 2003). In experimental models, periodontal bacteria have been shown to accelerate platelet aggregation, foam‐cell formation, and the development of atheroma (Lalla, Lamster, & Hofmann, 2003; Li, Messas, Batista Jr, Levine, & Amar, 2002). Intensive periodontal treatment has been shown to improve endothelial function (Tonetti, DAiuto, Nibaji, et al., 2007). A recent randomized clinical trial also revealed that periodontal treatment improved the endothelial function of patients following myocardial infarction (Lobo et al., 2020). *Porphyromonas gingivalis*, a major periodontopathic pathogen, might be involved in exacerbation of cardiovascular disease (Bugueno, Zobairi El‐Ghazouani, et al., 2020). A narrative review indicated that periodontal treatment may improve endothelial dysfunction, although the issue remains contentious (Parenti, Paccosi, Cairo, & Defraia, 2015).

Reactive hyperemia‐peripheral arterial tonometry (RH‐PAT) is an evaluation tool for vascular endothelial function, and allows assessment of the condition of arterial vascular function in just 15 min, regardless of artery size. Specifically, the ratio of arterial blood flow volume before and after release of blood flow is expressed as a numerical value, reactive hyperemia index (RHI). RH‐PAT offers a simpler approach than traditional methods such as flow‐mediated dilation (FMD) and was considered a useful tool in a previous study(Suzuki et al., 2014).

We hypothesized that vascular endothelial function is reduced in patients with periodontitis. The purpose of this study was to assess the relationship between periodontal condition and vascular endothelial function using RH‐PAT.

## 2 MATERIAL AND METHODS

### 2.1 Study population

Thirty‐three subjects were recruited from patients at Yokosuka Tower Clinic between September 2017 and March 2018. A physician picked out those who met the inclusion/exclusion criteria. Inclusion criteria were 20 years old or older and consent to participate in this study. Exclusion criteria were as follows: history and/or presence of other infection; regular administration of drugs resulting in gingival hyperplasia (such as cyclosporine and phenytoin) within the past 2 months; antibiotic intake within the past 2 months; dental visits within the past 6 months; or pregnancy.

The Ethics Committee of the School of Dentistry in Kanagawa Dental University approved the protocol for the present study (approval no. 436), and the protocol conformed to the 1975 Declaration of Helsinki, as revised in 2013. Written informed consent was provided by each participant.

### 2.2 Medical examination

Vascular endothelial function was examined using RH‐PAT (EndoPAT; Itamar Medical, Caesarea, Israel), as previously described (Kuvin et al., 2003). Briefly, patients could drink only water before the examination and stopped taking drugs, and did not take a bath or exercise on the morning of the test. During measurement, patients stayed in bed for 15 min under a steady environment to relax, representing no noise and comfortable temperature. The left arm was used as control, while the right arm was used as the test side. Measurement comprised a 5‐min baseline recording, 5 min of right brachial artery occlusion, and 5 min of RHI measurement. Brachial artery occlusion was performed using a sphygmomanometer cuff inflated to at least 60 mmHg over the systolic blood pressure of the patient. All analyses were performed in strict accordance with the instructions from the manufacturer.

General information and medical history of subjects such as age, sex, height, weight, blood pressure, smoking history, and serum hemoglobin (Hb) A1c level were collected from the medical records.

### 2.3 Clinical periodontal examination

A trained periodontist (NA) counted the number of teeth and recorded probing pocket depth (PPD), clinical attachment level (CAL) and bleeding on probing (BOP) at six points (buccal‐mesial, mid‐buccal, buccal‐distal, lingual‐mesial, mid‐lingual, and lingual‐distal) on all teeth including third molars with a manual probe (DISPON probe; BSA Sakurai Co., Nagoya, Japan). To avoid bias, the periodontist was blinded to medical records. Tooth mobility was examined using tweezers (DISPON pincet; BSA Sakurai Co.). To assess tooth mobility, Miller's classification was used as follows: 0, only slight, physiological movement when force was applied; 1, movement less than 1 mm in buccolingual or mesiodistal direction; 2, movement of 1 mm or more in the buccolingual and/or mesiodistal direction without vertical mobility; or 3, movement of 2 mm or more in the buccolingual and mesiodistal directions or in the vertical direction (Laster, Laudenbach, & Stoller, 1975; Miller, 1950). O'Leary's plaque control record (PCR) was scored at four sites (buccal, lingual, mesial, and distal), as previously described (O'Leary, Drake, & Naylor, 1972).

### 2.4 Data analysis

Results are shown as mean ± *SD* for continuous variables and as percentage for categorical variables. We divided subjects into two groups according to median RHI score. The low‐RHI group, with endothelial dysfunction, included participants with median RHI or lower (<1.45), while the high‐RHI group included all others. Comparison between groups was performed using Student's *t* test for continuous data and the *χ* ^2^ test for categorical data. Spearman's rank correlation coefficient was calculated to compare RHI scores and clinical periodontal parameters. Multivariate analysis was performed, using logistic regression. For this analysis, we used models that included age, sex, HbA1c, hypertension, dyslipidemia, and smoking status. JMP version 9.0.3 (SAS Institute Inc., Cary, NC) was used for all statistical analyses. Values of *p* < .05 were considered significant.

## 3 RESULTS

The characteristics of subjects in this study are shown in Table 1. No singificant differences in sex, smoking rate, body mass index (BMI), blood pressure, or frequencies of dyslipidemia, retinopathy, nephropathy, or neurological disorders were seen between groups. The low‐RHI group with endothelial dysfunction showed significantly higher mean age and lower HbA1c than the high‐RHI group.

**TABLE 1:** Characteristics of subjects

Variable	High RHI	Low RHI	*p*
Number	16	17	‐
RHI	1.80 ± 0.45	1.29 ± 0.13	<.001
Female [*n*, (%)]	9 (56)	10 (59)	.88
Age	55.1 ± 15.4	68.7 ± 12.4	.009
HbA1c [%]	7.58 ± 1.54	6.69 ± 0.59	.035
Smoke			
Never [*n*, (%)]	9 (56)	9 (53)	.17
Former [*n*, (%)]	3 (19)	7 (41)	‐
Current [*n*, (%)]	4 (25)	1 (6)	‐
BMI [kg/m^2^]	26.4 ± 5.8	25.2 ± 5.4	.56
SBP [mmHg]	136.2 ± 17.2	136.4 ± 16.6	.97
DBP [mmHg]	82.3 ± 15.0	77.0 ± 7.4	.20
Diabetes mellitus [*n*, (%)]	16 (100)	17 (100)	‐
Hypertension [*n*, (%)]	5 (31)	11 (65)	.052
Dyslipidemia [*n*, (%)]	9 (56)	6 (35)	.23
Retinopathy [*n*, (%)]	2 (13)	0 (0)	.082
Kidney disease [*n*, (%)]	1 (6)	2 (12)	.58
Neuropathy [*n*, (%)]	2 (13)	2 (12)	.95
Number of missing teeth	6.56 ± 4.23	12.06 ± 0.41	.040
PPD [mm]	2.32 ± 0.25	2.50 ± 0.52	.22
CAL [mm]	2.75 ± 0.63	3.19 ± 0.70	.075
BOP [%]	17.1 ± 11.6	22.3 ± 22.8	.42
Plaque control record [%]	45.2 ± 17.6	42.0 ± 22.7	.66
Patients with loosening teeth [*n*, (%)]	3 (19)	10 (63)	.010
Number of loosening teeth	0.38 ± 0.89	3.19 ± 3.54	.005
Sum of tooth mobility	0.38 ± 0.89	3.65 ± 3.82	.002

*Note:* Data are shown as mean ± *SD* or number and percentage. Comparison between groups was performed using Student's *t* test for continuous data and the *χ* ^2^ test for categorical data.

Abbreviations: BMI, body mass index; BOP, bleeding on probing; CAL, clinical attachment level; DBP, diastolic blood pressure; HbA1c, hemoglobin A1c; PPD, probing pocket depth; RHI, reactive hyperemia index; SBP, systolic blood pressure.

**FIGURE 1:**
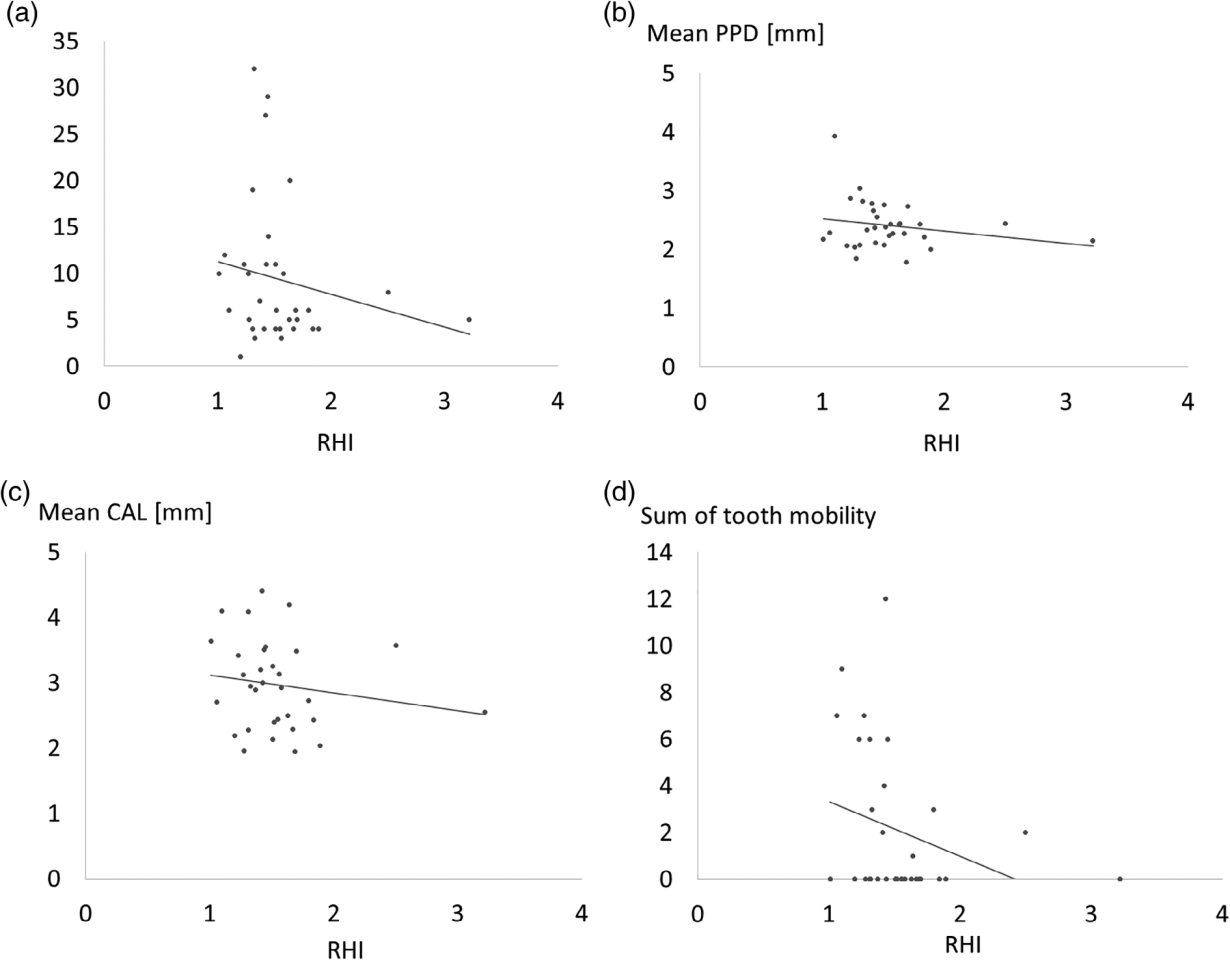
Association between each periodontal score and reactive hyperemia index (RHI). (a) Number of lost teeth and RHI. Spearman's rank correlation coefficient = −0.22, *p* = .21. (b) Probing pocket depth (PPD) and RHI. Spearman's rank correlation coefficient = −0.10, *p* = .59. (c) Clinical attachment level (CAL) and RHI. Spearman's rank correlation coefficient = −0.21, *p* = .25. (d) Total tooth mobility and RHI. Spearman’s rank correlation coefficient = −0.37, *p* = .035

Periodontal conditions of subjects are also shown in Table 1. Mean number of missing teeth was increased in the low‐RHI group. Mean PPD, CAL, BOP rate and PCR were comparable between groups. Many participants had loosened teeth and the mean number of loosened teeth was increased in the low‐RHI group. Total tooth mobility score was significantly increased in the low‐RHI group in comparison to the high‐RHI group. Figure 1 shows the association between each periodontal score and RHI. Total tooth mobility score was associated with RHI.

We performed multiple logistic regression analysis to assess whether patients with low RHI exhibited increased tooth mobility regardless of confounding factors such as age or HbA1c. High tooth mobility showed a relationship with low RHI after adjusting for age and HbA1c (Table 2). However, the association between RHI and tooth mobility was weaker in the fully adjusted model.

**TABLE 2:** Multiple logistic regression analysis for low reactive hyperemia index (RHI) score

	Unadjusted	Model 1	Model 2
Tooth mobility (Ref = No)	*p* = .010* OR 7.22 [1.44; 36.22]	*p* = .043* OR 6.92 [1.06; 45.21]	*p* = .059 OR 13.29 [0.90; 195.50]
HbA1c (Ref <7%)	‐	*p* = .051 OR 0.13 [0.02; 1.01]	*p* = .084 OR 0.086 [0.01; 2.19]
Age (Ref <65 years)	‐	*p* = .090 OR 5.39 [0.77; 37.68]	*p* = .17 OR 6.24 [0.46; 85.26]
Male sex (Ref = Female)	‐	‐	*p* = .35 OR 0.27 [0.02; 4.11]
Smoker	‐	‐	‐
Never	‐	‐	‐
Former	‐	‐	*p* = .29 OR 4.35 [0.28; 67.32]
Current	‐	‐	*p* = .38 OR 0.16 [0.00; 9.02]
Hypertension (Ref = No)	‐	‐	*p* = .72 OR 1.73 [0.09; 32.82]
Dyslipidemia (Ref = No)	‐	‐	*p* = .30 OR 0.26 [0.02; 3.32]

*Note: p‐*values, odds ratios (ORs) and 95% confidence intervals are shown. *p < .05. In Model 1, an association between endothelial function and tooth mobility was assessed with adjustment for age and hemoglobin (Hb) A1c. In Model 2, the association between endothelial function and tooth mobility is assessed in with adjustment for age, sex, HbA1c, hypertension, dyslipidemia and smoking status.

## 4 DISCUSSION

To the best of our knowledge, this is the first report to indicate an association between periodontitis and vascular endothelial function using the noninvasive RH‐PAT medical device. A relationship between tooth mobility and endothelial dysfunction was found (Table 1). Tooth mobility is one of the major symptoms in periodontitis.

Previous reports have shown associations between endothelial function as judged using other methods and systemic inflammation, cardiovascular diseases, and periodontal disease (Amar et al., 2003; Kitta et al., 2009; Kusunose et al., 2016; Matsuzawa, Sugiyama, Sumida, et al., 2013; Moura et al., 2017; Orlandi et al., 2014; Osmenda, Maciag, Wilk, et al., 2017; Suzuki et al., 2014; Tonetti et al., 2007). Several methods have been applied in the assessment of endothelial function, with FMD as one of the major tests of endothelial function (Matsuzawa, Guddeti, Kwon, Lerman, & Lerman, 2015). While FMD is a traditional method for assessing blood flow, RH‐PAT evaluates the ratio of arterial blood flow volume before and after the release of blood flow. RHI score from RH‐PAT has been statistically associated with the results of FMD(Kitta et al., 2009; Kuvin et al., 2003; Matsuzawa et al., 2013). Vascular analysis by RH‐PAT has advantages of easy usage and noninvasive features (Wilk et al., 2013). Several tests have been used for judging arterial stiffness, such as carotid‐femoral pulse wave velocity, branchial‐ankle pulse wave velocity, cardio‐ankle vascular index, and stiffness parameter β. The present study used RH‐PAT because of its strong point as a noninvasive method for measuring endothelial function. RH‐PAT is not useful for assessing large numbers of people, but its inspection accuracy and reproducibility have been recognized with use of a special probe (Kuvin et al., 2003).

The possibility of improved endothelial function by periodontal therapy has been shown (Kajikawa et al., 2014; Tonetti et al., 2007). Those reports suggested that periodontal infection and inflammation may be risk factors for endothelial dysfunction. Periodontitis was also indicated to induce endothelial dysfunction and treatment for severe periodontitis may reverse endothelial dysfunction (Lobo et al., 2020; Orlandi et al., 2014; Punj, Shenoy, & Subramanyam, 2017; Seinost et al., 2005). Subjects with severe periodontitis showing endothelial dysfunction and systemic inflammation are possibly at increased risk of cardiovascular disease (Amar et al., 2003; Libby, 1995; Moura et al., 2017) Smoking may play a role as a confounding factor (Kokubo et al., 2017), and an association between periodontal disease and endothelial dysfunction was indicated in smoking patients (Velosa‐Porras et al., 2016). A relationship between oral hygiene and endothelial function has also been found (Kajikawa et al., 2014). Periodontal treatment may be extremely useful, because improved endothelial function leads to prevention of atherogenesis and future cardiovascular events (Sen et al., 2018).

Reactive oxygen species (ROS) have been implicated in the pathogenesis of vascular diseases (Abid, Kachra, Spokes, & Aird, 2000; Kobayashi et al., 2003). The ROS system was originally regarded as a defense against exogenous microorganisms. A balance of ROS and antioxidant defense systems has also been implicated in the pathogenesis of periodontitis (Liu et al., 2017). Periodontal bacteria can easily disseminate into the systemic circulation through gingival injuries, leading to systemic inflammation (Hirschfeld & Kawai, 2015; Osmenda et al., 2017). Periodontal infection can promote ROS generation and enhanced oxidative stress in local and systemic inflammatory responses might contribute to the pathogenesis of cardiovascular diseases. Further investigations using animal models and interventional studies are needed to clarify the mechanisms underlying between periodontal disease and vascular diseases.

Several limitations to this study warrant consideration. Participants were recruited from a private medical hospital and most had type 2 diabetes mellitus. Periodontal condition varied markedly and a few cases of severe periodontitis cases were included. The high‐RHI group showed increased HbA1c (Table 1) and HbA1c level was associated with endothelial dysfunction. Each patient had received specific diabetic treatment and treatment period varied, which might have affected the results. However, tooth mobility correlated with endothelial function after adjustment for age and HbA1c. In considering generalizability, assessments of endothelial function and periodontal condition may be warranted in nondiabetes patients. Next, the number of participants in the present study was 33, representing a relatively small cohort. Although this was a pilot study, we should add data and confirm the results of this study in further investigations. Moreover, we used median RHI to divide patients into two groups in this study, because no consensus has been reached regarding cutoff points for RHI. As a result, a relationship between tooth mobility and endothelial function was found using this cutoff point; however, confirmation of the cutoff point should be performed in future studies.

In this study, an association between increased tooth mobility and endothelial dysfunction was found using RH‐PAT. Medical and dental cooperation may extend life expectancy, and control of oral infection may lead to prevention of noncommunicable diseases. Further investigations are needed to assess whether periodontal treatment can improve endothelial function and prevent cardiovascular outcomes.

## CONFLICT OF INTEREST

None.

## References

[cre2312-bib-0001] Abid, M. R. , Kachra, Z. , Spokes, K. C. , & Aird, W. C. (2000). NADPH oxidase activity is required for endothelial cell proliferation and migration. FEBS Letters, 486, 252–256. 11119713 10.1016/s0014-5793(00)02305-x

[cre2312-bib-0002] Amar, S. , Gokce, N. , Morgan, S. , Loukideli, M. , Van Dyke, T. E. , & Vita, J. A. (2003). Periodontal disease is associated with brachial artery endothelial dysfunction and systemic inflammation. Arteriosclerosis, Thrombosis, and Vascular Biology, 23, 1245–1249. 10.1161/01.ATV.0000078603.90302.4A 12763762

[cre2312-bib-0003] Beck, J. D. , Elter, J. R. , Heiss, G. , Couper, D. , Mauriello, S. M. , & Offenbacher, S. (2001). Relationship of periodontal disease to carotid artery intima‐media wall thickness: The atherosclerosis risk in communities (ARIC) study. Arteriosclerosis, Thrombosis, and Vascular Biology, 21, 1816–1822. 10.1161/hq1101.097803 11701471

[cre2312-bib-0004] Bugueno, I. M. , Zobairi El‐Ghazouani, F. , Batool, F. , Itawi, E. H. , Angles‐Cano, E. , Benkirane‐Jessel, N. , … Huck, O. (2020). *Porphyromonas gingivalis* triggers the shedding of inflammatory endothelial microvesicles that act as autocrine effectors of endothelial dysfunction. Scientific Reports, 10, 1778. 32019950 10.1038/s41598-020-58374-z PMC7000667

[cre2312-bib-0005] Hirschfeld, J. , & Kawai, T. (2015). Oral inflammation and bacteremia: Implications for chronic and acute systemic diseases involving major organs. Cardiovascular & Hematological Disorders Drug Targets, 15, 70–84. 25567334 10.2174/1871529x15666150108115241

[cre2312-bib-0006] Kajikawa, M. , Nakashima, A. , Maruhashi, T. , Iwamoto, Y. , Iwamoto, A. , Matsumoto, T. , … Higashi, Y. (2014). Poor oral health, that is, decreased frequency of tooth brushing, is associated with endothelial dysfunction. Circulation Journal, 78, 950–954. 24500034 10.1253/circj.cj-13-1330

[cre2312-bib-0007] Kitta, Y. , Obata, J. , Nakamura, T. , Hirano, M. , Kodama, Y. , Fujioka, D. , … Kugiyama, K. (2009). Persistent impairment of endothelial vasomotor function has a negative impact on outcome in patients with coronary artery disease. Journal of the American College of Cardiology, 53, 323–330. 19161880 10.1016/j.jacc.2008.08.074

[cre2312-bib-0008] Kobayashi, S. , Inoue, N. , Ohashi, Y. , Terashima, M. , Matsui, K. , Mori, T. , … Yokoyama, M. (2003). Interaction of oxidative stress and inflammatory response in coronary plaque instability: Important role of C‐reactive protein. Arteriosclerosis, Thrombosis, and Vascular Biology, 23, 1398–1404. 10.1161/01.ATV.0000081637.36475.BC 12805076

[cre2312-bib-0009] Kokubo, Y. , Watanabe, M. , Higashiyama, A. , Nakao, Y. M. , Kusano, K. , & Miyamoto, Y. (2017). Development of a basic risk score for incident atrial fibrillation in a Japanese general population—The Suita study. Circulation Journal, 81, 1580–1588. 28539563 10.1253/circj.CJ-17-0277

[cre2312-bib-0010] Kusunose, K. , Sato, M. , Yamada, H. , Saijo, Y. , Bando, M. , Hirata, Y. , … Sata, M. (2016). Prognostic implications of non‐invasive vascular function tests in high‐risk atherosclerosis patients. Circulation Journal, 80, 1034–1040. 26936237 10.1253/circj.CJ-15-1356

[cre2312-bib-0011] Kuvin, J. T. , Patel, A. R. , Sliney, K. A. , Pandian, N. G. , Sheffy, J. , Schnall, R. P. , … Udelson, J. E. (2003). Assessment of peripheral vascular endothelial function with finger arterial pulse wave amplitude. American Heart Journal, 146, 168–174. 12851627 10.1016/S0002-8703(03)00094-2

[cre2312-bib-0012] Lalla, E. , Lamster, I. B. , & Hofmann, M. A. (2003). Oral infection with a periodontal pathogen accelerates early atherosclerosis in apolipoprotein E‐null mice. Arteriosclerosis, Thrombosis, and Vascular Biology, 23, 1405–1411. 10.1161/01.ATV.0000082462.26258.FE 12816879

[cre2312-bib-0013] Laster, L. , Laudenbach, K. W. , & Stoller, N. H. (1975). An evaluation of clinical tooth mobility measurements. Journal of Periodontology, 46, 603–607. 1058939 10.1902/jop.1975.46.10.603

[cre2312-bib-0014] Li, L. , Messas, E. , Batista, E. L., Jr. , Levine, R. A. , & Amar, S. (2002). *Porphyromonas gingivalis* infection accelerates the progression of atherosclerosis in a heterozygous apolipoprotein E‐deficient murine model. Circulation, 105, 861–867. 11854128 10.1161/hc0702.104178

[cre2312-bib-0015] Libby, P. (1995). Molecular bases of the acute coronary syndromes. Circulation, 91, 2844–2850. 7758192 10.1161/01.cir.91.11.2844

[cre2312-bib-0016] Libby, P. , Ridker, P. M. , & Maseri, A. (2002). Inflammation and atherosclerosis. Circulation, 105, 1135–1143. 11877368 10.1161/hc0902.104353

[cre2312-bib-0017] Liu, C. , Mo, L. , Niu, Y. , Li, X. , Zhou, X. , & Xu, X. (2017). The role of reactive oxygen species and autophagy in periodontitis and their potential linkage. Frontiers in Physiology, 8, 439. 28690552 10.3389/fphys.2017.00439 PMC5481360

[cre2312-bib-0018] Lobo, M. G. , Schmidt, M. M. , Lopes, R. D. , Dipp, T. , Feijó, I. P. , Schmidt, K. E. S. , … Quadros, A. S. (2020). Treating periodontal disease in patients with myocardial infarction: A randomized clinical trial. European Journal of Internal Medicine, 71, 76–80. 31810741 10.1016/j.ejim.2019.08.012

[cre2312-bib-0019] Loos, B. G. , Craandijk, J. , Hoek, F. J. , Wertheim‐van Dillen, P. M. , & van der Velden, U. (2000). Elevation of systemic markers related to cardiovascular diseases in the peripheral blood of periodontitis patients. Journal of Periodontology, 71, 1528–1534. 11063384 10.1902/jop.2000.71.10.1528

[cre2312-bib-0020] Matsuzawa, Y. , Guddeti, R. R. , Kwon, T. G. , Lerman, L. O. , & Lerman, A. (2015). Secondary prevention strategy of cardiovascular disease using endothelial function testing. Circulation Journal, 79, 685–694. 25740088 10.1253/circj.CJ-15-0068

[cre2312-bib-0021] Matsuzawa, Y. , Sugiyama, S. , Sumida, H. , Sugamura, K. , Nozaki, T. , Ohba, K. , … Ogawa, H. (2013). Peripheral endothelial function and cardiovascular events in high‐risk patients. Journal of the American Heart Association, 2, e000426. 24275629 10.1161/JAHA.113.000426 PMC3886751

[cre2312-bib-0022] Miller, S. C. (1950). Textbook of Periodontia (p. 91). Philadelphia: Blakiston Co.

[cre2312-bib-0023] Moura, M. F. , Navarro, T. P. , Silva, T. A. , Cota, L. O. M. , Soares Dutra Oliveira, A. M. , & Costa, F. O. (2017). Periodontitis and endothelial dysfunction: Periodontal clinical parameters and levels of salivary markers interleukin‐1β, tumor necrosis factor‐α, matrix metalloproteinase‐2, tissue inhibitor of metalloproteinases‐2 complex, and nitric oxide. Journal of Periodontology, 88, 778–787. 28492359 10.1902/jop.2017.170023

[cre2312-bib-0024] Noack, B. , Genco, R. J. , Trevisan, M. , Grossi, S. , Zambon, J. J. , & De Nardin, E. (2001). Periodontal infections contribute to elevated systemic C‐reactive protein level. Journal of Periodontology, 72, 1221–1227. 11577954 10.1902/jop.2000.72.9.1221

[cre2312-bib-0025] O’Leary, T. J. , Drake, R. B. , & Naylor, J. E. (1972). The plaque control record. Journal of Periodontology, 43, 38. 4500182 10.1902/jop.1972.43.1.38

[cre2312-bib-0026] Orlandi, M. , Suvan, J. , Petrie, A. , Donos, N. , Masi, S. , Hingorani, A. , … D’Aiuto, F. (2014). Association between periodontal disease and its treatment, flow‐mediated dilatation and carotid intima‐media thickness: A systematic review and meta‐analysis. Atherosclerosis, 236, 39–46. 25014033 10.1016/j.atherosclerosis.2014.06.002

[cre2312-bib-0027] Osmenda, G. , Maciag, J. , Wilk, G. , Maciag, A. , Nowakowski, D. , Loster, J. , … Czesnikiewicz‐Guzik, M. (2017). Treatment of denture‐related stomatitis improves endothelial function assessed by flow‐mediated vascular dilation. Archives of Medical Science, 13, 66–74. 28144257 10.5114/aoms.2017.64715 PMC5206372

[cre2312-bib-0028] Parenti, A. , Paccosi, S. , Cairo, F. , & Defraia, E. (2015). Treatment of periodontitis for the prevention of endothelial dysfunction: A narrative review. Current Vascular Pharmacology, 13, 749–758. 26282351 10.2174/1570161113666150818110653

[cre2312-bib-0029] Pihlstrom, B. L. , Michalowicz, B. S. , & Johnson, N. W. (2005). Periodontal diseases. Lancet, 366, 1809–1820. 16298220 10.1016/S0140-6736(05)67728-8

[cre2312-bib-0030] Punj, A. , Shenoy, S. B. , & Subramanyam, K. (2017). Comparison of endothelial function in healthy patients and patients with chronic periodontitis and myocardial infarction. Journal of Periodontology, 88, 1234–1243. 28708039 10.1902/jop.2017.160748

[cre2312-bib-0031] Scannapieco, F. A. , Bush, R. B. , & Paju, S. (2003). Associations between periodontal disease and risk for atherosclerosis, cardiovascular disease, and stroke: A systematic review. Annals of Periodontology, 8, 38–53. 14971247 10.1902/annals.2003.8.1.38

[cre2312-bib-0032] Seinost, G. , Wimmer, G. , Skerget, M. , Thaller, E. , Brodmann, M. , Gasser, R. , … Pilger, E. (2005). Periodontal treatment improves endothelial dysfunction in patients with severe periodontitis. American Heart Journal, 149, 1050–1054. 15976787 10.1016/j.ahj.2004.09.059

[cre2312-bib-0033] Sen, S. , Giamberardino, L. D. , Moss, K. , Morelli, T. , Rosamond, W. D. , Gottesman, R. F. , … Offenbacher, S. (2018). Periodontal disease, regular dental care use, and incident ischemic stroke. Stroke, 49, 355–362. 29335336 10.1161/STROKEAHA.117.018990 PMC5780242

[cre2312-bib-0034] Slade, G. D. , Ghezzi, E. M. , Heiss, G. , Beck, J. D. , Riche, E. , & Offenbacher, S. (2003). Relationship between periodontal disease and C‐reactive protein among adults in the Atherosclerosis Risk in Communities study. Archives of Internal Medicine, 163, 1172–1179. 12767953 10.1001/archinte.163.10.1172

[cre2312-bib-0035] Slade, G. D. , Offenbacher, S. , Beck, J. D. , Heiss, G. , & Pankow, J. S. (2000). Acute‐phase inflammatory response to periodontal disease in the US population. Journal of Dental Research, 79, 49–57. 10690660 10.1177/00220345000790010701

[cre2312-bib-0036] Suzuki, H. , Matsuzawa, Y. , Konishi, M. , Akiyama, E. , Takano, K. , Nakayama, N. , … Kimura, K. (2014). Utility of noninvasive endothelial function test for prediction of deep vein thrombosis after total hip or knee arthroplasty. Circulation Journal, 78, 1723–1732. 24770356 10.1253/circj.cj-13-1325

[cre2312-bib-0037] Tonetti, M. S. , DAiuto, F. , Nibali, L. , Donald, A. , Storry, C. , Parkar, M. , … Deanfield, J. (2007). Treatment of periodontitis and endothelial function. The New England Journal of Medicine, 356, 911–920. 17329698 10.1056/NEJMoa063186

[cre2312-bib-0038] Velosa‐Porras, J. , Escobar‐Arregoces, F. , Latorre‐Uriza, C. , Ferro‐Camargo, M. B. , Ruiz, A. J. , & Uriza‐Carrasco, L. F. (2016). Association between periodontal disease and endothelial dysfunction in smoking patients. Acta Odontológica Latinoamericana, 29, 29–35. 27701495

[cre2312-bib-0039] Wilk, G. , Osmenda, G. , Matusik, P. , Nowakowski, D. , Jasiewicz‐Honkisz, B. , Ignacak, A. , … Guzik, T. J. (2013). Endothelial function assessment in atherosclerosis: Comparison of brachial artery flow‐mediated vasodilation and peripheral arterial tonometry. Polskie Archiwum Medycyny Wewnętrznej, 123, 443–452. 24025637 10.20452/pamw.1879

